# c-Jun N-Terminal Kinases (JNKs) in Myocardial and Cerebral Ischemia/Reperfusion Injury

**DOI:** 10.3389/fphar.2018.00715

**Published:** 2018-07-05

**Authors:** Maria Shvedova, Yana Anfinogenova, Elena N. Atochina-Vasserman, Igor A. Schepetkin, Dmitriy N. Atochin

**Affiliations:** ^1^Cardiovascular Research Center, Cardiology Division, Massachusetts General Hospital, Harvard Medical School, Charlestown, MA, United States; ^2^Cardiology Research Institute, Tomsk National Research Medical Center, Russian Academy of Sciences, Tomsk, Russia; ^3^RASA Center in Tomsk, Tomsk Polytechnic University, Tomsk, Russia; ^4^RASA Center, Kazan Federal University, Kazan, Russia; ^5^Department of Microbiology and Immunology, Montana State University, Bozeman, MT, United States

**Keywords:** brain, heart, c-Jun-N-terminal kinase, JNK inhibitor, ischemia/reperfusion injury, stroke

## Abstract

In this article, we review the literature regarding the role of c-Jun N-terminal kinases (JNKs) in cerebral and myocardial ischemia/reperfusion injury. Numerous studies demonstrate that JNK-mediated signaling pathways play an essential role in cerebral and myocardial ischemia/reperfusion injury. JNK-associated mechanisms are involved in preconditioning and post-conditioning of the heart and the brain. The literature and our own studies suggest that JNK inhibitors may exert cardioprotective and neuroprotective properties. The effects of modulating the JNK-depending pathways in the brain and the heart are reviewed. Cardioprotective and neuroprotective mechanisms of JNK inhibitors are discussed in detail including synthetic small molecule inhibitors (AS601245, SP600125, IQ-1S, and SR-3306), ion channel inhibitor GsMTx4, JNK-interacting proteins, inhibitors of mixed-lineage kinase (MLK) and MLK-interacting proteins, inhibitors of glutamate receptors, nitric oxide (NO) donors, and anesthetics. The role of JNKs in ischemia/reperfusion injury of the heart in diabetes mellitus is discussed in the context of comorbidities. According to reviewed literature, JNKs represent promising therapeutic targets for protection of the brain and the heart against ischemic stroke and myocardial infarction, respectively. However, different members of the JNK family exert diverse physiological properties which may not allow for systemic administration of non-specific JNK inhibitors for therapeutic purposes. Currently available candidate JNK inhibitors with high therapeutic potential are identified. The further search for selective JNK3 inhibitors remains an important task.

## Introduction

Studying the mechanisms of ischemia/reperfusion injury has a significant clinical relevance. Cerebral ischemia/reperfusion injury induces an increase in transendothelial permeability and blood-brain barrier damage in patients with stroke (Diaz-Cañestro et al., 2018; Liu et al., 2018). Cardiac ischemia/reperfusion is associated with necrosis, cardiomyocyte apoptosis, contractile dysfunction, and life-threatening ventricular arrhythmias (Girn et al., 2007; Monassier, 2008; Sharma et al., 2012). Ischemia/reperfusion injury may also essentially impact outcomes of vascularized tissue allotransplantation. Ischemia/reperfusion injury results in abnormal tissue architecture, hypertrophy of the cell nuclei, and powerful neovascularization in the presence of mitochondrial degeneration. Muscle cell necrosis is associated with a diffuse inflammatory infiltrate and vasculopathy continuing for hours after exposure; nerves undergo Wallerian degeneration (Messner et al., 2016). Recent studies have shown that the c-Jun N-terminal kinase (JNK) pathway is involved in ischemia/reperfusion injury (Ip and Davis, 1998; Nijboer et al., 2010; Javadov et al., 2014) as well as in neuronal apoptosis, tumor growth, and insulin resistance (Ji et al., 2015).

JNKs belong to a family of mitogen-activated protein kinases (MAPKs), which are activated in response to various stress stimuli such as ultraviolet radiation, oxidative stress, heat and osmotic shock, and ischemia/reperfusion injury of the brain and the heart (Knight and Buxton, 1996; Ip and Davis, 1998; Armstrong, 2004; Bogoyevitch and Kobe, 2006; Duplain, 2006; Bode and Dong, 2007). Multifunction and cooperation of MAPK signaling pathways is essential in other organs and tissues, in particular in eye wound healing and ischemic neuropathy (Luo et al., 2016; Yao et al., 2017). The family of JNK includes 10 isoforms encoded by three genes: JNK1 (four isoforms), JNK2 (four isoforms), and JNK3 (two isoforms) (Gupta et al., 1996; Waetzig and Herdegen, 2005). JNK1 and JNK2 are found in all cells and tissues of the body, while JNK3 is expressed mainly in the heart, brain, and testicles (Bode and Dong, 2007). JNK involved in pathogenesis of many diseases such as stroke, atherosclerosis, Alzheimer's and Parkinson's diseases (Waetzig and Herdegen, 2005; Johnson and Nakamura, 2007). Previous studies have shown that JNKs play an important role in the regulation of inflammation, the signaling pathways of apoptosis and necrosis and regulate several transcriptional and non-transcriptional processes involved in the injury of neurons and cardiomyocytes during ischemia and reperfusion (Ip and Davis, 1998; Nijboer et al., 2010; Javadov et al., 2014). JNKs also participate in the embryonic myocardial development, metabolic regulation, and other physiological processes including neuronal and immunological functions involving gene expression, cytoskeleton dynamics, and cell survival (Javadov et al., 2014).

Upstream, JNKs are activated by the MAP 2 kinases MAPK kinase (MKK) 3, MKK4, and MKK7, which, in turn, are activated by MAP 3 kinases (MKKKs), mixed lineage kinase (MLK)-2, MLK-3, transforming growth factor-β-activated kinase-1 (TAK-1), tumor progression locus-2 (Tpl2) kinase, and apoptosis signal-regulating kinase-1 (ASK1) (Guo et al., 2018). Upstream kinases of MAPK cascade (MKK4 and MKK7) phosphorylate and activate JNKs. The MAPK phosphatase (MKP) family represents essential regulators of JNKs. Being members of the cysteine-dependent dual-specificity protein phosphatase (DUSP) family, they specifically regulate the phosphorylation and activity of mammalian MAPKs (Guo et al., 2018).

Approximately a hundred of well-verified JNK substrates are now recognized. Among others, they comprise nuclear transcription factors (ATF2, c-Jun, Elk1, Myc), cytoplasmic proteins regulating cytoskeleton dynamics (DCX, Tau, WDR62), vesicular transporters or INK-interacting proteins JIP1/JIP3, transmembrane receptors (for example, bone morphogenetic protein receptor type 2, BMPR2), and mitochondrial proteins (Mcl1, Bim) (Zeke et al., 2016). Transcription factors such as c-Jun, activating transcription factor 2 (ATF2), Sp1, and nuclear factors of activated T-cells (NFATc2 and NFATc3) are substrates for phosphorylation-activated JNKs (Ip and Davis, 1998; Vlahopoulos and Zoumpourlis, 2004; Nijboer et al., 2010). There are also numerous non-nuclear substrates of JNKs, participating in the degradation of proteins, signal transduction, and regulation of apoptotic cell death (Bogoyevitch and Kobe, 2006; Shao et al., 2006). Dephosphorylation by dual specificity protein phosphatase (DUSP1/MKP-1) causes deactivation of the kinase (Chaudhury et al., 2010). Folding proteins, such as JNK-interacting proteins JIP-1 and Sab, and interaction with organelles are essential for the regulation of JNK activity (Wiltshire et al., 2004).

The heart and the brain are the two vital organs where ischemia/reperfusion injury plays the most crucial role causing the highest mortality and morbidity burden on the society. This review focuses on the involvement of JNK signaling in the pathophysiology of cerebral and myocardial ischemia/reperfusion injury and the emerging approaches to protect the heart and the brain.

## JNK in the pathophysiology of ischemia/reperfusion injury

**Figure 1:**
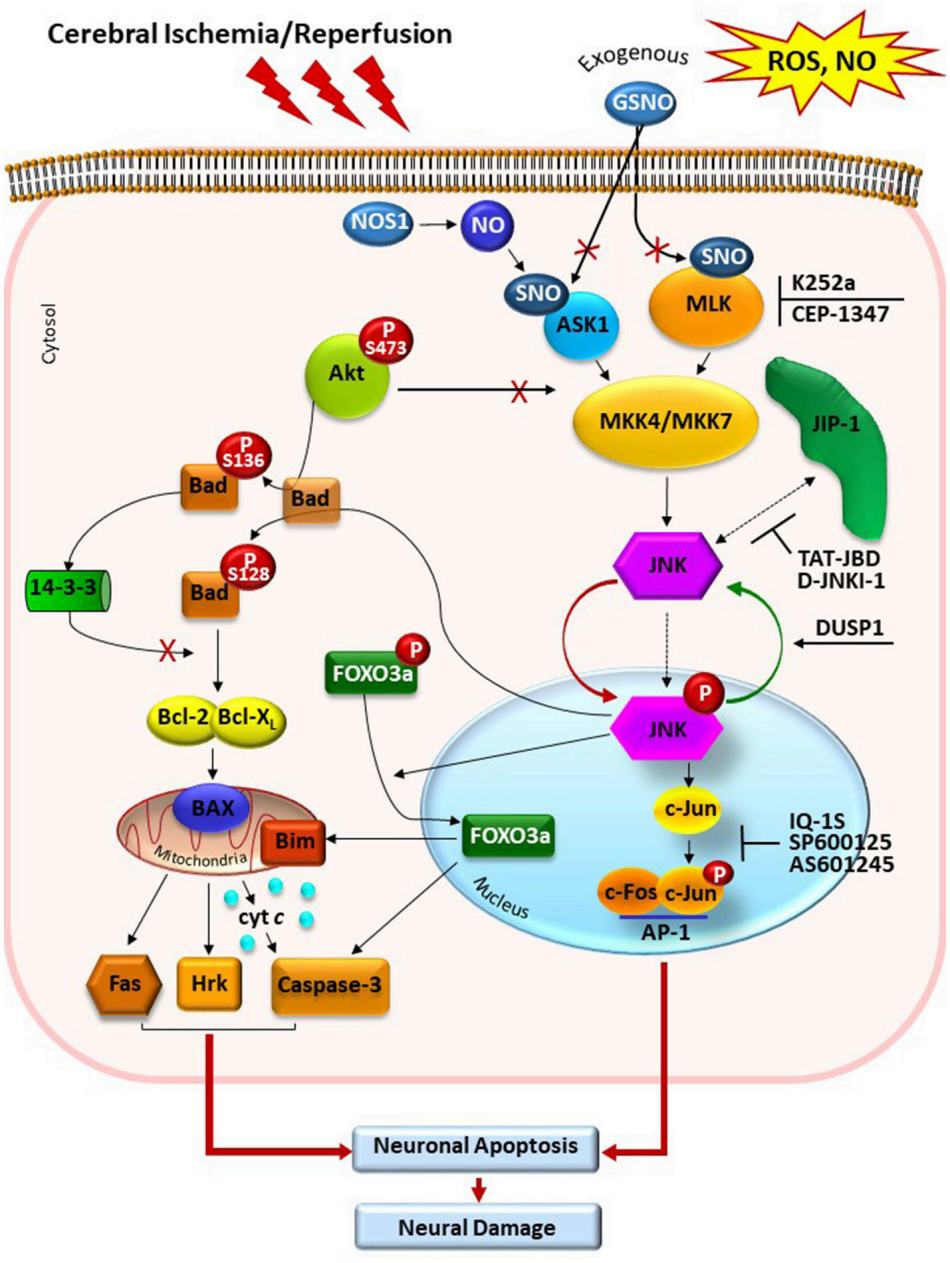
Schematic presentation of JNK signaling pathway in cerebral ischemia/reperfusion injury. Reactive oxygen species (ROS) and nitric oxide (NO) eruption after ischemia/reperfusion cause activation of MAP kinase kinases MLK3 and ASK1 that can phosphorylate MAP kinase kinases MKK4/7. Exogenous NO, generated by exogenous NO donors, S-nitrosoglutathione (GSNO) and endogenous NO, generated by NO synthases, modulates ASK1 and MLK3 activity via its S-nitrosylation. JNK dephosphorylation by DUSP1 causes deactivation of this MAPK. Following activation, JNK translocates to the nucleus and modulates the function of AP-1 transcription factor, which induces a change in gene transcription, resulting in biological responses such as inflammation and/or apoptosis. MLK inhibitors K252a and CEP-1347, and JNK inhibitors SP600125, IQ-1S, and AS601245, block activities of MLK and phosphorylated JNK, respectively. Other stimulus and effects in the figure are listed in the text. GSNO, S-nitrosoglutathione; NO, nitric oxide; NOS, NO synthase; ASK1, apoptosis signal-regulated kinase 1; MLK, mixed-lineage kinase; SNO, S-nitrosylated sites of the ASK1 and MLK; MKK4/7, MAP kinase kinases 4/7; JNK, c-Jun-terminal kinase; p-JNK, phosphorylated JNK; JIP-1, JNK-interacting protein; Akt, protein kinase B; Bad, Bcl-2-associated death promoter; DUSP1, dual specificity protein phosphatase; FOXO3a, transcription factor of Forkhead family.

**Figure 2:**
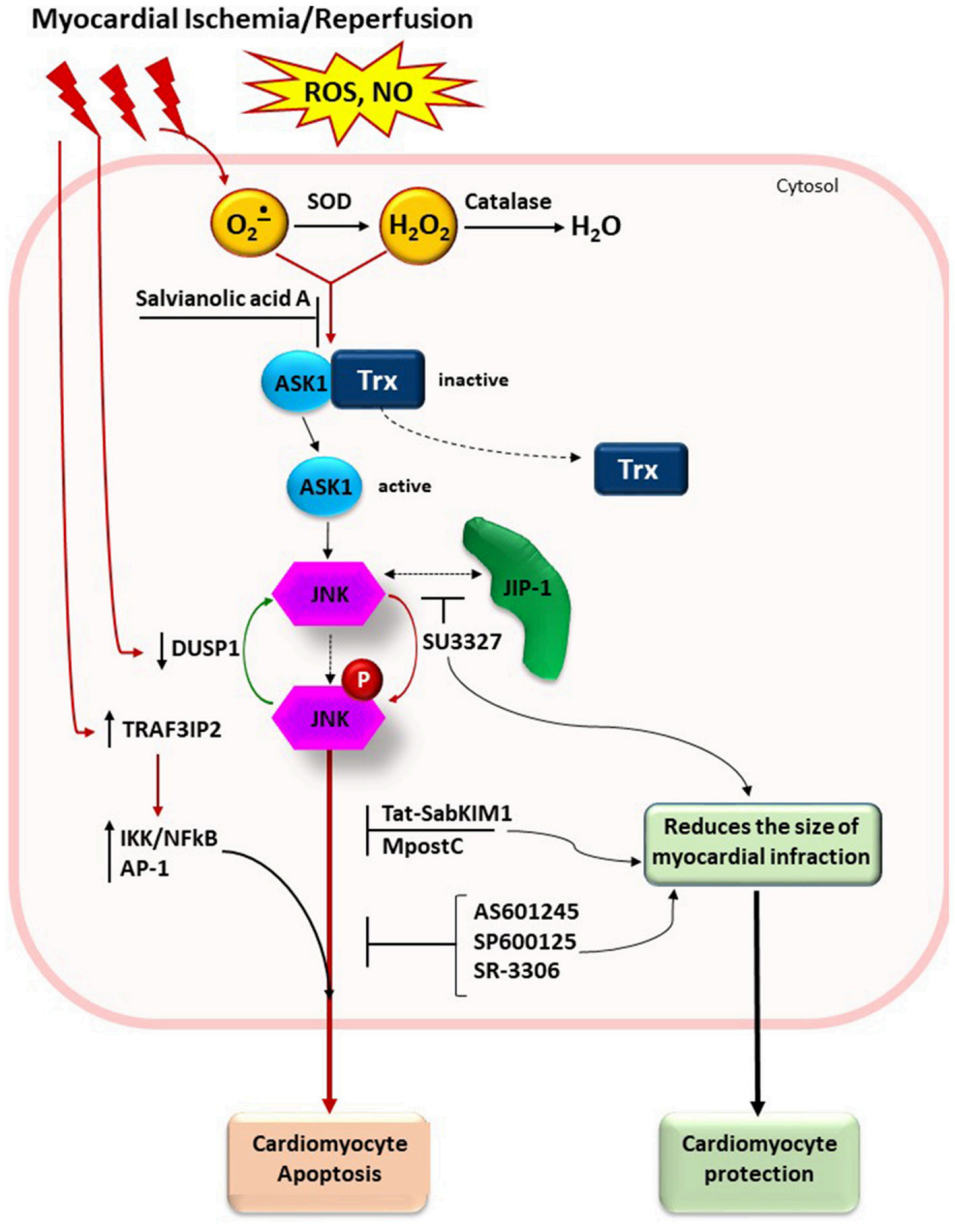
Schematic presentation of JNK signaling pathway in myocardial ischemia/reperfusion injury. ROS generation after ischemia/reperfusion leads to oxidation of SH groups in thioredoxin and its dissociation from the complex with ASK1. Antioxidant enzymes (SOD and catalase) and small molecules with free radical scavenging capacity (for example, salvianolic acid A) could attenuate the oxidative stress. JNK inhibitors SP600125, AS601245, and SR-3306 block activity of phosphorylated JNK. Small molecule inhibitor SU3327 blocks the process of spontaneous folding of polypeptide chain in the JNK-binding domain of JIP that also attenuates the JNK activation. Tat-SabKIM1, a retro-inverso peptide blocks interaction of JNK with mitochondria (not shown). Other stimulus and effects in the figure are listed in the text. SOD, superoxide dismutase; Trx, thioredoxin; ASK1, apoptosis signal-regulated kinase 1; JNK, c-Jun-terminal kinase; p-JNK, phosphorylated JNK; JIP-1, JNK-interacting protein; DUSP1, dual specificity protein phosphatase; MpostC, morphine postconditioning; TRAF3IP2, TRAF3 interacting protein 2.

The well-defined regulation of JNK signaling pathways in cerebral and myocardial ischemia/reperfusion injury are summarized in Figures 1, 2.

### JNK in cerebral ischemia/reperfusion injury

Here we update information on the significance of JNK-mediated signaling in the brain as previously discussed (Shvedova et al., 2017). JNKs are involved in many neuropathological events and also play role in physiological neuronal regulation (Kuan et al., 2003). JNK signaling pathway plays a critical role in mediating apoptosis following cerebral ischemia and reperfusion (Pei et al., 2008). Numerous works show that the increased JNK phosphorylation and activation of JNK-dependent pathway occur after global and focal cerebral ischemia in rats and mice (Hayashi et al., 2000; Irving and Bamford, 2002; Borsello et al., 2003; Ferrer et al., 2003; Tian et al., 2005; Atochin et al., 2016). Activation of JNK aggravates brain injury in stroke, provoking inflammation and leading to ischemic cell death (Davis, 2000). An important role of JNK in the mechanisms of neuronal death and survival is confirmed by the experiments with Jnk1−/− knockout mice where permanent middle cerebral artery occlusion significantly expands the area of the infarction with an increased expression of JNK3 in the penumbra (Brecht et al., 2005). Considering that the cells in the infarct area undergo necrosis, this observation does not contradict the fact that JNK promotes cell apoptosis.

Ferrer and co-authors studied the distribution of phosphorylated JNK (p-JNK) and its substrate c-Jun in the early stages after reperfusion in the rat model of middle cerebral artery occlusion (MCAO). The study showed that nuclear phosphorylation of JNK and c-Jun is increased 4 h after reperfusion in the central zone of the infarction; the increased level of their cytoplasmic phosphorylation is seen in the area between the intact and the infarction area (penumbra) (Ferrer et al., 2003).

One of the mechanisms by which activation of JNK contributes to neuronal apoptosis in ischemia and reperfusion consists in phosphorylation of Bcl-2-associated death promoter (Bad) associated with anti-apoptotic protein Bcl-2, induction of Bim and Fas proteins, caspase activation, as well as the release of cytochrome *c* from the mitochondria (Kuan et al., 2003; Qi et al., 2009). The study of JNK signaling pathway in *Cornu Ammo*nis area 1 (CA1) hippocampal neuronal apoptosis in gerbils showed activation of caspases −3, −8, and −9 in the first few days after transient cerebral ischemia as well as later release of cytochrome *c* from the mitochondria at days 5–7 after the ischemia (Carboni et al., 2005).

Proteins of Bcl-2 family play an important role in the regulation of apoptosis in global and focal cerebral ischemia (Zinkel et al., 2006; Wang et al., 2007; Rami et al., 2008). After transient global ischemia Bad protein is phosphorylated in the area of CA1 of the hippocampus. Protein kinase B (Akt)-dependent phosphorylation of Bad at serine 136 enhances the interaction of Bad with phosphopeptide-binding proteins 14-3-3 which prevent the dimerization of Bad with Bcl-XL, inhibits the release of cytochrome c into the cytosol, and suppresses the activation of caspase-3 that ultimately increases neuronal survival (Wang et al., 2007). Besides, activated Akt1 attenuates the phosphorylation of Bad at serine 128 by downregulating activation of JNK1/2, thus limiting JNK-dependent apoptosis. In contrast, phosphorylation of Bad at serine 128 after brain ischemia/reperfusion injury occurs due to the kinase activity of JNK1 and JNK2 (Wang et al., 2007). The phosphorylation suppresses Bad interaction with 14-3-3 proteins, which contributes to the apoptotic effect of Bad. It has been reported that Akt can phosphorylate ASK1 on Ser83, which results in the inhibition of apoptosis induced by ASK1 (Kim et al., 2001). Akt can also phosphorylate MLK3 on Ser674 and this causes MLK3 inactivation and the promotion of cell survival (Barthwal et al., 2003).

Dephosphorylation of JNK by DUSP1 leads to JNK deactivation (Keyse, 2008). It is expected that the DUSP1 can limit the increase in the JNK activity and reduce the death of neurons in hypoxia and reperfusion (Koga et al., 2012). JNK-interacting proteins JIP-1 and Sab play an important role in the regulation of JNK activity (Barr et al., 2004; Beeler et al., 2009). It should be noted that increased expression of JIP-1 is detected in the brain (Beeler et al., 2009).

### JNK in the myocardial ischemia/reperfusion injury

In this section of the review, we provide new details on the JNK-mediated signaling in the myocardium which we briefly presented before (Shvedova et al., 2016). JNK-dependent pathway is an important step in the pathological mechanisms of myocardial hypertrophy and ischemia/reperfusion injury of the heart (Javadov et al., 2014). JNK is activated in cardiac ischemia and reperfusion and may be involved in the protective and adverse processes in the myocardium (Fryer et al., 2001; Kaiser et al., 2005; Rose et al., 2010; Wei et al., 2011; Javadov et al., 2014). This activation of JNK is transient, but it may vary depending on the severity and timing of oxidative stress during ischemia and/or reperfusion (Knight and Buxton, 1996; Laderoute and Webster, 1997; He et al., 1999; Fryer et al., 2001; Armstrong, 2004). Both protective and harmful effects of JNK are shown in genetic models where Jnk1^−/−^ and Jnk2 ^−/−^ knockout mice or MAPK kinase 7 (MKK7) overexpression mice demonstrate protective effects on cardiomyocytes in ischemia/reperfusion-induced apoptosis *in vivo* (Kaiser et al., 2005). Despite the fact that activation of JNK is insignificant during myocardial ischemia due to coronary artery bypass surgery in humans, there is an increase of JNK activity in cardiac tissue during reperfusion (Talmor et al., 2000). Some effects of myocardial ischemia and reperfusion can be reproduced *in vitro* by placing cardiomyocytes in “ischemic” buffer and an oxygen-free atmosphere (usually 95% nitrogen and 5% carbon dioxide). This model of ischemia and subsequent reoxygenation induce an increase in the levels of phosphorylation and activity of JNK in neonatal H9c2 cardiomyocytes (He et al., 1999; Sun et al., 2012).

Chambers and co-authors showed that JNK-dependent signaling leads to the generation of reactive oxygen species (ROS), mitochondrial dysfunction, and loss of cardiomyocytes (Chambers et al., 2013). It has been shown that sepsis can induce processes similar to ischemic preconditions. Bacterial lipopolysaccharide exposure protects isolated cardiomyocytes against hypoxia-induced cell death via JNK-associated signaling pathways (Walshe et al., 2015). Activation of JNKs significantly contributes to myocardial ischemia/reperfusion injury during heart transplantation (Vassalli et al., 2012). Recent data demonstrated that JNK is involved in suppressing the proliferation of mesenchymal stem cells. Considering that the mesenchymal stem cells play an important role in recovery of the heart from post-ischemic injury (Wu et al., 2011), prevention of ischemia-mediated suppression of their proliferative activity using JNK inhibitors may have therapeutic value for healing the ischemic lesions.

JNKs are involved in the regulation of apoptosis of cardiomyocytes through the activation of caspase-dependent (Aoki et al., 2002) and caspase-independent pathways in the mitochondria (Song et al., 2008; Zhang G. M. et al., 2009). One of the pathophysiological mechanisms by which activation of JNK contributes to cardiomyocytes apoptosis in ischemia/reperfusion injury, is the regulation of Bad phosphorylation (Qi et al., 2009). Bcl-2 inhibits apoptosis in many cellular systems, including lymphohematopoietic and neuronal cells. Bcl-2 regulates cell death by affecting the permeability of the mitochondrial membranes and suppressing caspase through preventing the release of cytochrome *c* from the mitochondria and/or binding the apoptosis inducing factor (AIF) (Song et al., 2008). In the rat model of heart ischemia/reperfusion injury *in vivo*, administration of SP600125, an anthrapyrazolone inhibitor of JNK that competes with ATP to inhibit the phosphorylation of c-Jun, attenuates mitochondria-to-nuclear translocation of AIF, cardiomyocyte apoptosis, and the size of necrosis (Song et al., 2008; Zhang J. et al., 2009). The key processes involved in the activation of apoptosis via JNK-dependent pathway occur in the mitochondria. In general, functioning of the mitochondrial proteins can be modulated through either phosphorylation or nitrosylation, or through the changes in the protein localization. Activation of mitochondrial JNK, rather than localization of this enzyme to the mitochondria, contributes to autophagy and apoptosis exacerbating the subsequent myocardial injury (Xu et al., 2015). JNK activation in the mitochondria during ischemia/reperfusion requires entry of Ca^2+^, the movement of electrons to the electron transport chain proteins on the inner membranes of the mitochondria, and ROS generation (Dougherty et al., 2002, 2004). Thus, in the isolated rat heart perfused immediately prior to ischemia with Ca^2+^-free media, the activation of JNK is abolished (Knight and Buxton, 1996). However, JNK has high binding ability with the mitochondria through protein Sab. Moreover, pharmacological blocking of JNK binding with Sab reduces the size of infarction in rat hearts (Chambers et al., 2013). It should be noted that the activation of mitochondrial JNK could slow down the respiration and ATP production and thereby negatively affect the bioenergetic mitochondrial function (Dougherty et al., 2004).

ROS could be generated by NADPH-oxidase, electron-transport protein chains of the mitochondria, or they could arise from other sources (Dougherty et al., 2002, 2004; Oshikawa et al., 2012; Khalid et al., 2016). ROS generation leads to activation of JNK and protein kinase C (Frazier et al., 2007). The introduction of H_2_O_2_ to the perfusion solution activates JNK in the isolated heart, although this activation is less pronounced than in the ischemia/reperfusion model (Clerk et al., 1998). On the other hand, the introduction of catalase and superoxide dismutase (SOD) to the perfusion solution suppresses the activation of JNK in cardiomyocytes (Knight and Buxton, 1996). In some models, the activation of JNK can support generation of ROS. Adaptor protein p66Shc-dependent ROS production via JNK-dependent activation of NADPH-oxidase contributes to numerous pathways including ischemia/reperfusion injury (Oshikawa et al., 2012; Khalid et al., 2016). Application of JNK inhibitor SP600125 significantly reduces p66Shc phosphorylation at serine 36 in HL-1 cardiomyocytes in ischemia/reperfusion model (Khalid et al., 2016). Thus, JNK inhibitors could prevent activation of p66Shc and subsequent oxidative stress.

JNK activation by ROS could be mediated via ASK1, which is the upstream redox sensor of ROS generation in the MAPK cascade with a high affinity binding to a reduced form of redox-responsive protein thioredoxin, which prevents dimerization and activation of ASK1 (Soga et al., 2012). Oxidative stress promotes oxidation and dissociation of thioredoxin from the complex and autophosphorylation of ASK1 (Kaminskyy and Zhivotovsky, 2014).

Cardio-specific protein MuRF1 regulates the size of cardiomyocytes through its ubiquitin ligase activity, which contributes to the subsequent degradation of sarcomere proteins, as well as through interaction with transcription factors that are involved in the molecular mechanisms of cardiac hypertrophy (Li et al., 2011). Cardio-protective properties of MuRF1 in myocardial ischemia/reperfusion are due to suppression of JNK signal transduction pathways via proteasome-dependent degradation of activated JNKs, as well as decreasing cardiomyocytes apoptosis (Li et al., 2011). In contrast, other ubiquitin ligase atrogin-1 causes a sustained activation of JNKs through a mechanism that involves degradation of MAPK phosphatase-1 (MKP-1) protein that leads to apoptosis of cardiomyocytes after ischemia/reperfusion. SP600125 blocks the effect of atrogin-1 on cell apoptosis and the expression of apoptotic-related proteins and caspases (Xie et al., 2009).

JNK-associated mechanisms are involved in many regulatory pathways of inflammation. Activated protein C (APC) is a vitamin K-dependent plasma serine protease that downregulates blood clotting and inflammatory pathways (Wang et al., 2011). It is known that the APC exerts a cardioprotective effect by decreasing JNK activity, reducing apoptosis of cardiomyocytes, and suppressing expression of inflammatory mediators after myocardial ischemia (Aoki et al., 2002). Likewise, nuclear protein high-mobility group box 1 (HMGB1) is involved in the myocardial inflammation and injury induced by ischemia/reperfusion. This protein acts in concert with the tumor necrosis factor (TNF) to promote ischemia/reperfusion-induced myocardial apoptosis though JNK activation. It has been shown that JNK inhibitor SP600125 prevents the cardiomyocyte apoptosis induced by TNF/HMGB1 *in vitro* (Xu et al., 2011). Additionally, macrophage migration inhibition factor (MIF) is a proinflammatory cytokine, which plays an important role in chronic inflammatory diseases. MIF reduces JNK activation during reperfusion and protects the heart from injury (Qi et al., 2009). Moreover, in the isolated heart of Mif ^−/−^ knockout mice there is an enhanced JNK activation (Qi et al., 2009). There is an assumption that, during ischemia/reperfusion, endogenous MIF expressed in the heart overwhelms JNK-dependent way through its specific receptor CD74 and 5'AMP-activated protein kinase (AMPK). Advanced glycation end-products (AGEs) bind to and trigger the receptor for AGEs (RAGE), which is one of the major modulators of inflammation. AGEs are also involved in the mechanisms of acute ischemia/reperfusion injury of the heart (Shang et al., 2010). Previously it has been shown that AGEs/RAGE interaction transduces signals through activation of JNK and other MAPKs, leading to activation of proapoptotic pathways and cardiomyocyte death under hypoxia/reperfusion (Shang et al., 2010). Finally, regulator of G-protein signaling 5 (RGS5) can inhibit the activity of JNK1/2. RGS5 is highly expressed in the human adult heart and is a guanosine triphosphatase-activating protein that inhibits many pathways promoting cardiomyocytes ischemia/reperfusion-induced apoptosis. This mechanism protects the cardiomyocytes from apoptosis during ischemia/reperfusion (Wang Z. et al., 2016).

JNK can also play a protective role. In particular, JNK activates Akt via its phosphorylation of threonine 450 in a post-ischemic injury (Shao et al., 2006). Decrease in Akt activity, caused by JNK inhibition, reduces the survival of isolated cardiomyocytes following hypoxia *in vitro* (Shao et al., 2006). These data demonstrate that JNK participates in post-ischemia Akt reactivation that might be the main mechanism of protective effect of JNK in cardiomyocytes (Shao et al., 2006). Protective role of JNK is also shown in cultural neonatal cardiomyocytes. Treatment of cells with SP600125 leads to activation of caspase-3 and subsequent apoptosis (Engelbrecht et al., 2004).

Therefore, signaling mechanisms of JNK-dependent pathways may adversely or positively affect the impact of other factors involved in ischemia/reperfusion heart injury. The interaction of JNK with other kinases, such as p38, AMPK, protein kinase C and Akt, plays an important role. Regulation of JNK activity involves Ca^2+^ ions, various regulatory proteins and ROS. Signaling molecules associated with JNK might be targets and effectors of these interactions. Pro- and anti-apoptotic effects of JNK in ischemia/reperfusion perhaps depend on the expression and activation of these kinases and regulatory proteins as well as on the intracellular redistribution of activated JNK between the cytoplasm, mitochondria, and the nucleus of the cardiomyocytes.

#### JNK in myocardial ischemic pre- and post-conditioning

The term “ischemic heart preconditioning” usually means short-term (transient) ischemia, which leads to increased resistance of the myocardium to injury associated with subsequent ischemia and reperfusion. A few sessions of transient ischemia and reperfusion exert cardioprotective effects (Maslov et al., 2013). Despite similarity in the molecular mechanisms of cardiac preconditioning and post-conditioning, several review papers stated JNK-dependent pathways differ between these processes (Hausenloy and Yellon, 2006; Maslov et al., 2013). In most experimental models, preconditioning induces activation of JNK (Ping et al., 1999; Hausenloy and Yellon, 2007), whereas post-conditioning is accompanied by the suppression of JNK activity (Sun et al., 2006; Li et al., 2009; Zhang G. M. et al., 2009). For example, cardiac preconditioning in rabbits activates the phosphorylation of JNK at two amino acid residues; there are important differences between p46 and p54 forms of JNK in their subcellular localization in the cardiomyocytes (cytoplasmic or nuclear fraction) and the mechanism of activation (ischemia or reperfusion). Activation of p46-JNK occurs during ischemia, whereas phosphorylation of amino residue 54 occurs after reperfusion (Ping et al., 1999). However, Nakano and co-authors were not able to detect JNK activation after ischemic preconditioning in the model of the isolated heart (Nakano et al., 2000). The cardioprotective effect of post-conditioning can be caused by suppression of JNK activity in the myocardium. A significant decrease in the level of phosphorylation is observed in various models of ischemic post-conditioning (Liu et al., 2006; Li et al., 2009; Zhang G. M. et al., 2009; Wei et al., 2015), including post-conditioning with gradually increased reperfusion (Zhang et al., 2014). Reduced phosphorylation of JNK also occurs in isolated cardiomyocytes in the simulated hypoxic post-conditioning model (Liu et al., 2008).

## Effects of modulation of JNK activity

Several synthetic inhibitors of enzymatic activity of JNK have been described, including small molecules SP600125, AS601245, IQ-1S, SR-3306; protein and non-protein molecules inhibiting the interactions between JNK and their substrates or between JNK and the folding proteins and/or cellular organelles have been proposed as well (Irving and Bamford, 2002; Carboni et al., 2004, 2005; Gao et al., 2005; Guan et al., 2005; Kuan and Burke, 2005; Pan et al., 2005; Krenitsky et al., 2012; Schepetkin et al., 2012; Gehringer et al., 2015). However, a search for highly selective JNK inhibitors, suitable for therapeutic purposes continues. Some of these molecules show neuroprotective effects in the animal models of stroke and myocardial infarction (Koch et al., 2015; Atochin et al., 2016). Structures of several small molecule inhibitors of JNK signaling pathway with therapeutic effects in models of myocardial and cerebral ischemia/reperfusion injury are shown in Table 1.

**Table 1:** Small molecule inhibitors of JNK signaling pathway with therapeutic effects in models of myocardial and cerebral ischemia/reperfusion injury.

**Name**	**Molecular structure**	**Molecular target/mechanism**	**References**
SP600125	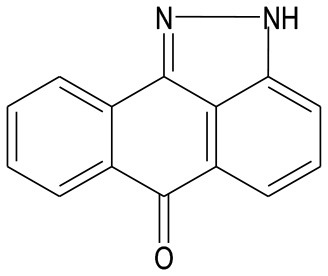	JNK inhibitor	Engelbrecht et al., 2004; Gao et al., 2005; Guan et al., 2005, 2006; Song et al., 2008; Shi et al., 2009; Xie et al., 2009; Zhang J. et al., 2009; Xu et al., 2011, 2015; Murata et al., 2012; Khalid et al., 2016; Shao et al., 2017
AS601245	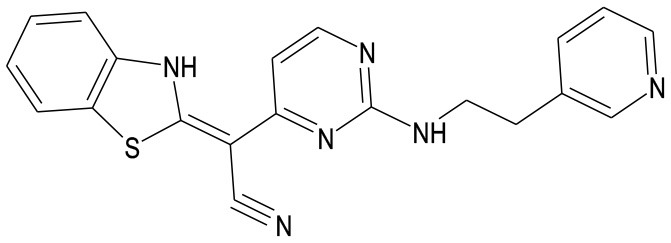	JNK inhibitor	Carboni et al., 2004, 2005, 2008; Li et al., 2015
IQ-1S	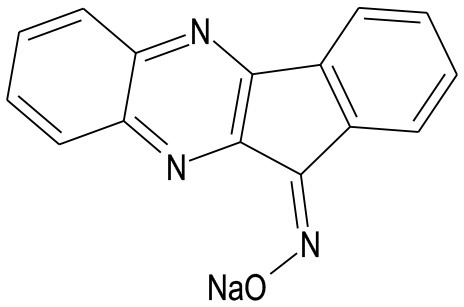	JNK inhibitor	Atochin et al., 2016
SR-3306	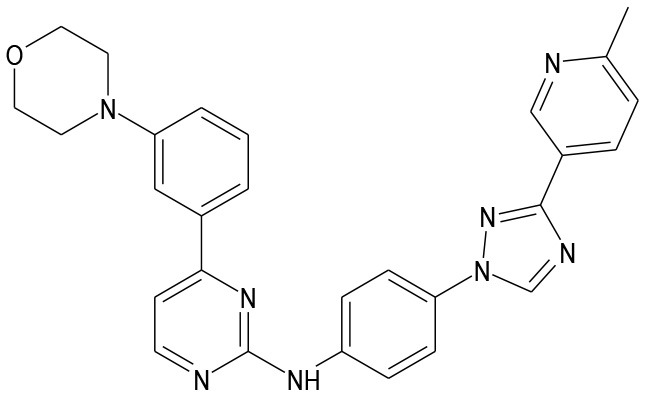	JNK inhibitor	Chambers et al., 2013
CEP-1347	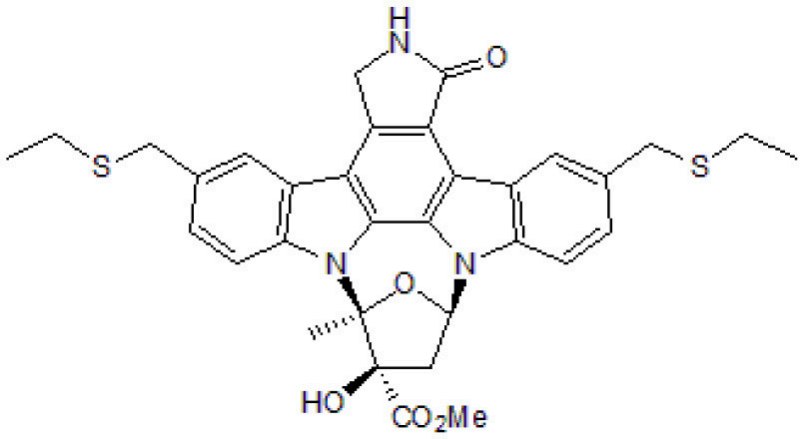	MLK inhibitor	Carlsson et al., 2009
SU3327	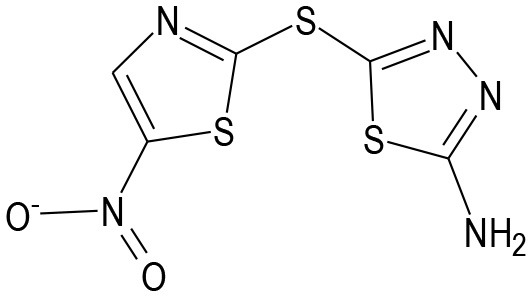	blocks the folding of polypeptide chain in the JNK-binding domain of JIP	Jang and Javadov, 2014

*Chemical names: SP600125, anthra[1-9-cd]pyrazol-6(2H)-one; AS601245, 1,3-benzothiazol-2-yl (2-{[2-(3-pyridinyl)ethyl]amino}-4 pyrimidinyl) acetonitrile; IQ-1S, 11H-indeno[1,2-b]quinoxalin-11-one oxime sodium salt; SR-3306; N-(4-(3-(6-methylpyridin-3-yl)-1H-1,2,4-triazol-1-yl)phenyl)-4-(3-morpholinophenyl)pyrimidin2-amine; CEP-1347, (9S,10R,12R)-5-16-bis[(ethylthio)methyl]-2,3,9,10,11,12-hexahydro-10-hydroxy-9-methyl-1-oxo-9,12-epoxy-1H-diindolo[1,2,3-fg:3′,2′,1′-kl]pyrrolo[3,4-i][1,6]benzodiazocine-10-carboxylic acid methyl ester; SU3327, 5-[(5-nitro-2-thiazolyl)thio]-1,3,4-thiadiazol-2-amine*.

### Inhibition of the JNK activity in the brain

#### Neuroprotective effects of small-molecule inhibitors of JNK activity

SP600125 is a cell penetrating inhibitor of JNK. This inhibitor shows neuroprotection property in various models of ischemia/reperfusion brain injury. The compound reduces neuronal apoptosis and infarction volume and improves stroke outcomes (Gao et al., 2005; Guan et al., 2005; Murata et al., 2012). SP600125 inhibits neuronal apoptosis induced by global ischemia followed by reperfusion in CA1 region of the hippocampus (Guan et al., 2006). SP600125 reduces phosphorylation of c-Jun and the expression of Fas ligand (FasL), induced by ischemia/reperfusion in the region CA1 of the hippocampus. It should be noted that caspase-3 activation caused by ischemia/reperfusion is also significantly inhibited by preliminary administration of SP600125. Similar neuroprotective effect is observed after administration of SP600125 before or after ischemia in rats. Thus, SP600125 can inhibit the activation of JNK and provide neuroprotection in ischemia/reperfusion in the area CA1 of the hippocampus by suppressing neuronal apoptosis (Guan et al., 2006).

Gao et al. studied JNK-dependent mechanism of apoptotic neuronal death after focal ischemia/reperfusion injury in mice (Gao et al., 2005). The researchers found an increase in the activity of JNK in the brain 0.5–24 h after ischemia. Activation of JNK induces serine phosphorylation of protein 14-3-3, which leads to its dissociation from the Bax complex and translocation of Bax to the mitochondria (Gao et al., 2005). The study also showed that systemic administration of SP600125 reduces the infarct volume and the ischemia-induced expression of Fas, Bim and protein Hrk from the Bcl-2 family; it prevents Bax and Bim translocation to the mitochondria, the release of cytochrome *c* and proapoptotic protein Smac, and the activation of the caspases−3 and−9 (Gao et al., 2005). It should be noted, however, that SP600125 is relatively nonspecific. For example, 13 of the 28 tested kinases were suppressed by this compound at the same or even greater degree compared with JNK (Bain et al., 2003). Therefore, it cannot be ruled out that the observed protective effects of SP600125 during ischemia/reperfusion are associated with the inhibition of other kinases.

Although JNK inhibition at early stage of ischemia/reperfusion reduces infarct volume and improves stroke outcomes, delayed inhibition of JNK activity could have the adverse effects on these parameters. For example, in ischemia/reperfusion model, administration of SP600125 10 min after the stroke reduces infarct volume, while the administration of this inhibitor 7 days after the stroke increases the volume of brain infarction and exacerbates neurological disorders. Immune staining of near-infarction zone in samples with delayed administration of SP600125 suggests the reduced expression of neurovascular remodeling markers, including matrix metalloproteinase-9 (MMP-9) in the astrocytes, as well as the reduction of microvascular density network. Thus, JNK, involved in neuronal death at the early stages, may also be involved in the endogenous processes of neurovascular remodeling and restoration after cerebral ischemia (Murata et al., 2012).

Another JNK inhibitor, AS601245, also contributes to the survival of cells after cerebral ischemia. The administration of this inhibitor (intraperitoneally, at doses of 40, 60, and 80 mg/kg) significantly reduces the loss of CA1 hippocampal neurons in the model of transient global cerebral ischemia in gerbils (Carboni et al., 2004). The administration of AS601245 at a high dose of 80 mg/kg reduces damage to neuronal processes and attenuates astrogliosis, contributing to the preservation of long-term memory in mice (Carboni et al., 2008). Significant neuroprotective effect of AS601245 administration is observed after intraperitoneal (6, 18, and 60 mg/kg) or intravenous (1 mg/kg) administration in rats after focal cerebral ischemia (Carboni et al., 2004). AS601245 protects hippocampal neurons in the CA1 area from cell death, counteracting the activation of both mitochondrial and non-mitochondrial pathways of apoptosis (Carboni et al., 2005).

In the model of cerebral hypoxia/ischemia in 7-day old rats, phosphorylation of JNK is accompanied by dephosphorylation of the transcription factor of Forkhead family (FOXO3a) that leads to the translocation of FOXO3a to the nucleus, which, in turn, increases the level of expression of proapoptotic protein Bim and caspase-3 (Li et al., 2015). Inhibition of JNK by intracerebroventricular injection of AS601245 30 min prior to cerebral hypoxia/ischemia leads to a significant increase in FOXO3a phosphorylation that attenuates the FOXO3a translocation to the nucleus after the injury. In addition, the inhibition of JNK reduces the levels of Bim and caspase-3, decreasing the neuronal apoptosis and the area of infarction in the brain of rats (Li et al., 2015).

Recently, a new JNK inhibitor with a dual action, IQ-1S, has been described. This inhibitor also releases nitric oxide (NO) as a result of bioconversion by microsomal oxidoreductases (Schepetkin et al., 2012; Atochin et al., 2016). Therapeutic efficacy of this JNK inhibitor has been studied in mice with experimental cerebral ischemia and reperfusion. Intraperitoneal administration of IQ-1S reduces neurological deficit and infarction volume in comparison with control animals 48 h after reperfusion (Atochin et al., 2016).

#### JNK-interacting proteins and indirect modulation of JNK-dependent pathway in the brain

Synthetically designed peptide TAT-JBD consists of a sequence of approximately 20 amino acids that corresponds to the JNK binding domain (JBD) of JNK interacting protein (JIP) coupled to the protein transduction sequence of HIV-TAT. TAT-JBD inhibits the ensemble of complex JNK with JIP-1, and therefore inhibits JNK activity. It should be noted that this sequence increases the ability of molecules to penetrate into the cell. Intraperitoneal administration of the TAT-JBD peptide immediately after hypoxia/ischemia prevents activation of the transcription factor AP-1 in rats (Nijboer et al., 2010). Administration of TAT-JBD during the first 3 h after hypoxia/ischemia reduces neuronal damage. The analysis of apoptotic cell death markers demonstrates that TAT-JBD significantly reduces hypoxia/ischemia-induced activity of caspase-3. However, administration of TAT-JBD does not inhibit other factors mediating apoptosis such as the activation of caspase-8, dissociation of Bid, and the release of mitochondrial cytochrome *c* (Nijboer et al., 2010). TAT-JBD suppresses hypoxia/ischemia-induced expression of Smac, an inhibitor of apoptosis proteins (IAP). Application of TAT-JBD also reduces cleavage of α-fodrin indicating a decrease in the calpain-mediated brain injury. Neuroprotection, induced by TAT-JBD administration, is long-lasting, as the damage to the gray and white matter of the brain is decreased by 50% at week 14 after hypoxia/ischemia along with the significant improvements in behavior and cognitive functioning of rats (Nijboer et al., 2010).

Signaling pathway of JNKs can also be suppressed by blocking the access to their protein substrates. One of such blockers is D-retro-inverse protein D-JNKI-1 also based on TAT peptide sequence. This inhibitor of JNK has been studied in several models *in vivo* (Borsello et al., 2003; Wiegler et al., 2008) and its neuroprotective properties have been demonstrated in animal models of cerebral ischemia and stroke (Deloche et al., 2014).

Some synthetic antioxidants, including propyl gallate and edaravone, show neuroprotective effects in the models of ischemia/reperfusion injury and their effects are associated with a reduction in the activity of JNK (Wen et al., 2006; Zheng et al., 2006).

The activation of the calcium-sensing receptor (CaSR), a G-protein coupled receptor, promotes apoptosis in focal cerebral ischemia/reperfusion in male adult Kunming mice subjected to 2-h focal cerebral ischemia followed by 22-h reperfusion, which may be related to the activation of JNK/p38 MAPK pathways. Ischemia/reperfusion increases CaSR expression and induces neuronal apoptosis in the brain. Gadolinium trichloride (GdCl_3_), an agonist of CaSR, further deteriorates neurological dysfunction, increases infarct volume, enhances CaSR expression, and promotes neuronal apoptosis. A low p-JNK expression is detected in the cortex and hippocampus of sham group, while p-JNK expression is significantly up-regulated after ischemia/reperfusion. GdCl_3_ could further enhance the p-JNK expression induced by ischemia/reperfusion, but p-JNK expression is drastically inhibited by NPS2390, an inhibitor of CaSR (Zhen et al., 2016).

In mouse cerebral ischemia/reperfusion injury model (2 h of transient MCAO, followed by 24 h of reperfusion) penehyclidine hydrochloride (PHC), a new cholinergic antagonist, significantly downregulates the phosphorylation of JNK, p38MAPK, and c-Jun and protects against cerebral ischemia/reperfusion injury. PHC-treated mouse group in comparison with control group shows improved neurological deficits, and blood-brain barrier integrity as well as reduced infarction volume, brain water content, and apoptosis (Shu et al., 2016).

Butylphthalide, which belongs to a family of compounds initially isolated from the seeds of *Apium graveolens*, administered intragastrically three times a day at a dosage of 15 mg/kg beginning at 20 min after brain ischemia/reperfusion in Sprague-Dawley rats, protects neurons against cerebral ischemia/reperfusion-induced damage remarkably improving the survival of CA1 pyramidal neurons in brain injury and inhibits the JNK/caspase-3 signaling pathway. The expression of p-JNK, p-Bcl-2, p-c-Jun, FasL, and cleaved caspase-3 is decreased in the rats treated with butylphthalide (Wen et al., 2016). Precise mechanisms of butylphthalide effects on JNK during cerebral ischemia/reperfusion remain unknown.

Neuropeptide, a calcitonin gene-related peptide (CGRP) improves the neurobehavioral function and reduces the cerebral infarction area in rats subjected to ischemia/reperfusion (MCAO model). Western blotting results confirm that the function of the CGRP is mediated mainly through the reduction of the JNK and p38 phosphorylation and the promotion of extracellular regulated kinase (ERK) phosphorylation (Yang et al., 2016).

Atorvastatin, a hypolipidemic agent protects hippocampal CA1 pyramidal neurons against cerebral ischemia/reperfusion in four-vessel occlusion model in rats. Atorvastatin could increase the phosphorylation of Akt1 and NO synthase diminish the phosphorylation of JNK3 and c-Jun, and further inhibit the activation of caspase-3. All of these protective effects of atorvastatin are reversed by LY294002 [an inhibitor of phosphatidylinositide 3-kinase (PI3K)]. Pretreatment with JNK inhibitor SP600125 diminishes the phosphorylation of JNK3 and c-Jun, and further inhibits the activation of caspase-3 after cerebral ischemia/reperfusion (Shao et al., 2017).

Anfibatide is a GPIb antagonist derived from the protein complex agglucetin. Anfibatide has protective effects against cerebral ischemia/reperfusion injury in rats in MCAO model. The underlying mechanism may be associated with the suppression of apoptosis through inhibiting toll-like receptor 4 (TLR4)/JNK/caspase-3 pathway (Luo et al., 2017).

Neuregulin-1β is a protein, which belongs to a four-member family of epidermal growth factor-like signaling molecules. Neuregulin-1β shows a neuroprotective effect through the JNK signaling pathway in rats in MCAO model of ischemia/reperfusion. Neuregulin-1β treatment decreases JNK activity and the protein levels of p-JNK, p-MKK4, and p-c-Jun. It also attenuates the ischemia-induced apoptosis and infarct volume and recovers the neurological function (Ji et al., 2017). Underlying mechanisms of neuregulin-1β-mediated decrease in JNK activity are not well understood.

#### Inhibitors of mixed-lineage kinase (MLK) and MLK-interacting proteins in the brain

Pharmacological modulation of JNK activity may be achieved by influencing the upstream elements of the signaling pathway. Brain ischemia causes prolonged activation of the MLK3/MKK7/JNK3 cascade (Pan et al., 2005). Introduction of a microbial-origin alkaloid K252a (Kase et al., 1986), a potent inhibitor of MLK3 and tyrosine protein kinase activities, 20 min prior ischemia inhibits MLK3/MKK7/JNK3 signaling, Bcl-2 phosphorylation, the activation of c-Jun and caspase-3 without significant effects on these protein expressions. K252a also significantly increases the number of the surviving CA1 pyramidal cells at 5 days of reperfusion. These data demonstrate that the alkaloid K252a plays a neuroprotective role in ischemic injury by inhibiting the signaling pathways of JNK involving apoptotic effector of caspase-3 (Pan et al., 2005).

Carlsson et al. also found that introduction of CEP-1347, a synthetic analog of K252a and a potent inhibitor of MLK family kinases has a protective effect due to suppression of apoptosis of hippocampal neurons in the model of neonatal hypoxia/ischemia (Carlsson et al., 2009). CEP-1347 also demonstrated neuroprotective properties *in vitro* by blocking JNK activation and subsequent events associated with the activation of the JNK pathway in the model of β-amyloid-induced apoptosis (Bozyczko-Coyne et al., 2001). It should be noted that CEP-1347 does not inhibit the enzymatic activity of JNK (Maroney et al., 1998).

Global cerebral ischemia with subsequent reperfusion in rat model of four-vessel occlusion ischemia can enhance the binding of heat shock protein hsp90 with MLK3 and thereby cause the activation of JNK3. Geldanamycin, an alkaloid of microbial origin, is an inhibitor of protein chaperon hsp90 (Bedin et al., 2004); hsp90 together with the p50cdc37 is required to activate MLK (Zhang et al., 2004). Geldanamycin reduces the expression of MLK3 protein and JNK activation and exhibits neuroprotection *in vivo* and *in vitro* (Wen et al., 2008). However, geldanamycin itself is an unfortunate drug candidate because its administration in physiological concentrations is associated with hepatotoxicity and eryptosis. This requires continuing development of geldanamycin analogs with better safety profiles.

#### Glutamate receptors inhibitors affect JNK activity in the brain

Kainate glutamate subtype receptor (GluR6) plays an important role in the ischemic brain through Glur6-PSD95-MLK3 signaling module, activation of kinases MKK4/7, and subsequent phosphorylation of JNK and apoptosis factors (Pei et al., 2006; Du et al., 2009; Di et al., 2012). It has been shown that the maximum assembly of GluR6-PSD95-MLK3 module occurs at 6 h of reperfusion (Tian et al., 2005). Pei and co-authors used antisense oligonucleotides to GluR6 to suppress the expression of these receptors. These oligonucleotides were intracerebraventricularly injected to rats (once a day for 3 days) the transient ischemia of the brain caused by four-vessel occlusion. The results showed that the oligonucleotides to GluR6 suppress the expression of these receptors, assembly of GluR6-PSD95-MLK3 signaling module, JNK activation, and c-Jun phosphorylation and, ultimately, protect neurons from death induced by ischemia/reperfusion (Pei et al., 2005). Interestingly, hypothermia (32°C) induced 10 min prior ischemia and maintained for 3 h after ischemia can also inhibit the assembly of the GluR6-PSD95-MLK3 signaling module and the activation of MLK3, MKK4/7, and JNK3. The hypothermia reduces phosphorylation of c-Jun and expression of FasL, reduces translocation of Bax, the release of cytochrome c, and caspase-3 activation in CA1 area of the hippocampus (Hu et al., 2008).

#### NO donors and JNK activity in the brain

More and more evidence suggests that JNK pathway is entangled with enzymatic production of NO by NO synthases, and subsequent S-nitrosylation of proteins during ischemia and reperfusion (Yu et al., 2008). In contrast, exogenous NO donors reduce S-nitrosylation of MLK3 protein caused by reperfusion and inhibit the activation of JNK-dependent pathway (Hu et al., 2012). NO donor, sodium nitroprusside, reduces JNK3 phosphorylation and damage of hippocampal neurons after global ischemia/reperfusion (Pei et al., 2008). Other kinases may be involved in NO-dependent modulation of JNK activity. For example, S-nitrosylation of ASK1 by endogenous NO activates JNK-dependent cascade during cerebral ischemia and reperfusion (Liu et al., 2013). At the same time, exogenous NO, generated by NO donors, such as sodium nitroprusside and S-nitrosoglutathione, suppresses S-nitrosylation of ASK1 and protects neurons during ischemia/reperfusion (Liu et al., 2013). Thus, exogenous NO donor can exert therapeutic effects through modulating the MLK and ASK1 activities.

#### Neuroprotective effects of anesthetics and JNK activity

It has been shown that propofol, an intravenous anesthetic and a modulator of ion channels TRPA1 and TRPA5 (Bahnasi et al., 2008; Sinha et al., 2015), inhibits neuronal apoptosis in ischemic stroke, protects the brain against ischemia/reperfusion injury, and enhances neural function. Ji et al. studied the effects of propofol (intravenous injection 30 min post reperfusion) in ischemia/reperfusion model with MCAO (Ji et al., 2015). They demonstrated that the levels of water and Evans blue (used for the evaluation of blood-brain barrier permeability to macromolecules) as well as the expression of MMP-9 and p-JNK in the brain are significantly reduced in propofol group (Ji et al., 2015).

Other studies showed that isoflurane, an anesthetic and a modulator of ion channel TRPA1 (Cornett et al., 2008), has neuroprotective effects against ischemia/reperfusion injury in the MCAO model. Isoflurane in concentrations up to 1.5% can regulate the expression of transforming growth factor-β1 (TGF-β1) and suppress the expression of p-JNK that significantly attenuates ischemia/reperfusion injury. This protective effect ceases when the signaling pathway of TGF-β1 is blocked by LY2157299, a specific inhibitor of tyrosine kinase receptor for TGF-β (Wang S. et al., 2016).

### Cardiac effects of inhibiting JNK activity

#### Synthetic inhibitors of JNK activity in the heart

Pharmacological inhibition of JNK by various synthetic inhibitors, such as AS601245, SP600125, and SR-3306 (Table 1), reduces the size of myocardial infarction and attenuates cardiomyocyte apoptosis after ischemia/reperfusion injury (Yue et al., 2000; Ferrandi et al., 2004; Duplain, 2006; Liu et al., 2007; Milano et al., 2007; Liu et al., 2008; Song et al., 2008; Shi et al., 2009, Zhang J. et al., 2009; Xu et al., 2011; Chambers et al., 2013; Khan et al., 2017). Introduction of SP600125 into the solution for perfusion of the isolated mouse heart prior to ischemia/reperfusion increases the resistance to opening the mitochondrial permeability transition pore and protects the myocardium against contractile dysfunction and necrosis during ischemia/reperfusion (Zaha et al., 2016). Pretreatment with SP600125 for 30 min improves survival of the cardiomyocytes after ischemia/reperfusion *in vitro* (Xie et al., 2009). This inhibitor also increases cardioprotective effect of insulin in ischemia/reperfusion injury (Liu et al., 2007). Application of selective inhibitor of JNK, SR-601245, 5 min before the end of ischemia protects the myocardium during ischemia/reperfusion in the experimental animal models. This inhibitor reduces infarct volume and attenuates ischemia/reperfusion-induced increases in the activity of creatine phosphokinase and creatine kinase in blood (Chambers et al., 2013). However, a dual inhibitor of MAPKs JNK and p38, compound V-150 when administered prior to ischemia worsens cardiomyocyte apoptosis and myocardial infarction in the animal model of prolonged ischemia (Shao et al., 2006) emphasizing the relevance of focusing on the effects of selective JNK inhibitors.

#### Effects of indirect modulation of JNK activity in the heart

Indirect modulation of JNK activity in the heart may be a promising approach for cardioprotection. For example, small molecule inhibitor SU3327 (see Table 1) blocks the process of spontaneous folding of polypeptide chain in the JNK-binding domain of JIP (Jang and Javadov, 2014). The presence of this inhibitor in the perfusion solution improves cardiac performance of the isolated rat hearts and reduces myocardial injury after ischemia/reperfusion (Jang and Javadov, 2014). Moreover, inhibition of JNK1 expression in the cardiomyocytes *in vitro* by using antisense oligonucleotides protects against ischemia-induced apoptosis while silencing JNK2 does not produce similar effect (Hreniuk et al., 2001).

Another example is a D enantiomer of a spider venom peptide GsMTx4 (selective inhibitor of cationic mechanosensitive ion channels), which shows cardioprotective effects in a mouse model of ischemia/reperfusion. Administration of the GsMTx4-D reduces infarct area and inhibits JNK/c-Jun, but also inhibits the energy response Akt signaling system (Wang J. et al., 2016).

Interestingly, xanthine oxidase and xanthine dehydrogenase have been shown to be implicated in production of myocardial damage following reperfusion of an occluded coronary artery. Febuxostat and allopurinol (xanthine oxidase inhibitors) pretreatment exerts cardioprotective effects in rats subjected to one-stage left anterior descending coronary artery ligation for 45 min followed by a 60-min reperfusion. Suppression of the active JNK and p38 proteins with the rise in ERK1/ERK2 is more prominent after pretreatment with febuxostat in comparison with allopurinol pretreatment (Khan et al., 2017).

Sodium hydrosulfide, NaHS, a donor of hydrogen sulfide (H_2_S), is also a promising agent. In a simulated ischemia/reperfusion model with primary cultured rat neonatal cardiomyocytes, ischemia/reperfusion induces a rapid, time-dependent JNK phosphorylation with significant elevation at 15 and 30 min during reperfusion. Treatment with H_2_S significantly inhibits the early phosphorylation of JNK, especially at 15 min. Both NaHS and JNK inhibitor SP600125 decrease the number of apoptotic cells in a simulated ischemia/reperfusion model. However, if NaHS application is delayed by 1 h after reperfusion, the inhibition of apoptosis is negated (Shi et al., 2009).

Moreover, treatment with Tat-SabKIM1, a retro-inverso peptide which blocks interaction of JNK with mitochondria, decreases mitochondrial JNK activation without changing JNK mitochondrial localization following reperfusion. Tat-SabKIM1 treatment reduces Bcl2-regulated autophagy, apoptosis, and myocardial infarct size (Chambers et al., 2013; Xu et al., 2015). Selective inhibition of mitochondrial JNK activation using Tat-SabKIM1 peptide produces an infarct size-reducing effect similar to inhibiting JNK with SP600125 (Xu et al., 2015).

Genetic modulation of signaling molecules upstream of JNK has been used in the experiments to elucidate the JNK-associated regulatory cascades. Cardiac DUSP1 is downregulated following acute cardiac ischemia/reperfusion injury. DUSP1 transgenic mice (DUSP1TG mice), compared to wild-type mice, demonstrate a smaller infarcted area and the improved myocardial function *in vivo*. The ischemia/reperfusion-induced DUSP1 deficiency promotes the activation of JNK which upregulates the expression of the mitochondrial fission factor (Mff). The loss of DUSP1 amplifies the Bnip3 phosphorylated activation via JNK, leading to the activation of mitophagy. While the reintroduction of DUSP1 blunts Mff/Bnip3 activation and alleviates the fatal mitochondrial fission/mitophagy by inactivating the JNK pathway, providing a survival advantage to myocardial tissue following ischemia/reperfusion stress (Jin et al., 2017).

TRAF3IP2 (TRAF3 interacting protein 2; previously known as CIKS or Act1) is an oxidative stress-responsive cytoplasmic adapter molecule that is an upstream regulator of both IκB kinase (IKK) and JNK. Ischemia/reperfusion up-regulates TRAF3IP2 expression in the heart, and its gene deletion, in a conditional cardiomyocyte-specific manner, significantly attenuates ischemia/reperfusion-induced oxidative stress, IKK/NF-κB and JNK/AP-1 activation, inflammatory cytokine, chemokine, and adhesion molecule expression, immune cell infiltration, myocardial injury, and contractile dysfunction (Erikson et al., 2017).

#### Anesthesia modulates JNK activity in the heart

Morphine post-conditioning (MpostC) markedly reduces infarct size, creatine kinase MB isozyme release and improves cardiac function recovery in isolated rat hearts subjected to ischemia/reperfusion injury via inhibiting the phosphorylation of JNK and p38 kinases, mitochondrial permeability transition pore opening and cytochrome *c* release. These protective effects are partially abolished in the presence of anisomycin (an activator of JNK/p38 kinases) that totally reverses the inhibitory effects of MpostC on the phosphorylation of JNK/p38 kinases, permeability transition pore opening, and cytochrome *c* release. However, when used individually, anisomycin does not influence perfusion injury (Chen Z. et al., 2016).

### Pharmacological modulation of JNK activity in diabetes protects the brain and the heart

Metabolic abnormalities represent one of the most essential risk factors for myocardial infarction and stroke. Patients with diabetes mellitus have worse clinical outcomes after acute ischemic stroke (Rosso et al., 2015). The presence of diabetes mellitus significantly modulates adaptive responses and tolerance to ischemia and reperfusion. Glucagon-like peptide-1 (GLP-1) is an incretin hormone that increases glucose-dependent insulin secretion resulting in the reduction of the glucose level. This molecule is involved in the pathogenesis of type 2 diabetes mellitus due to defective glucose-stimulated insulin secretion. Intracerebroventricular administration of GLP-1 receptor agonist, exendin-4, after cerebral ischemia/reperfusion injury reduces infarct volume in rats. JNK signaling is inhibited by 36% within 24 h after exendin-4 injection. Islet-brain 1, a scaffold regulator of JNK, is increased by 1.7-fold by exendin-4 (Kim et al., 2017). A long-lacting GLP-1 analog, liraglutide, exerts neuroprotective action in the experimental models. Liraglutide inhibits cell apoptosis by attenuating excessive ROS and improving the mitochondrial function in the neurons suffering oxygen glucose deprivation *in vitro* and *in vivo*. Liraglutide down-regulates the phosphorylation of JNK and p38 and augments the phosphorylation of Akt and ERK. Liraglutide demonstrates therapeutic potential for patients suffering from ischemic stroke, especially comorbid with type 2 diabetes mellitus or stress hyperglycemia (Zhu et al., 2016).

Insulin selectively inhibits mitochondrial JNK activation, contributing to the cardioprotective effects of insulin against ischemia/reperfusion. A new antidiabetic drug, rosiglitazone (a peroxisome proliferator-activated receptor (PPAR)-γ agonist) is associated with suppressed JNK phosphorylation in cardiac tissue of both normal and diabetes animals (Khandoudi et al., 2002). In rat model of myocardial ischemia/reperfusion, treatment with rosiglitazone significantly reduces myocardial infarction in mice treated with rosiglitazone compared with vehicle controls (Morrison et al., 2011). When rosiglitazone is administrated simultaneously with onset of reperfusion, JNK-dependent inflammatory response is inhibited, which greatly improves recovery of the myocardium after ischemia/reperfusion injury (Morrison et al., 2011). Insulin selectively inhibits the activation of mitochondrial JNK, protecting the myocardium against ischemia/reperfusion injury (Xu et al., 2015). Insulin simultaneously activates both Akt and JNK, and the latter further increases the phosphorylation of Akt which attenuates ischemia/reperfusion injury and improves cardiac function (Liu et al., 2007). Thus, the cross-talk between Akt and JNK is involved in insulin-induced cardioprotection.

Metformin exerts direct protective effects against high-glucose and hypoxia/reoxygenation injury in H9C2 rat cardiomyoblasts via signaling mechanisms involving activation of AMPK and suppression of high-glucose and hypoxia/reoxygenation-induced JNK activation. Inhibitor of AMPK (compound C) and activator of JNK (anisomycin) abolish the protective effects of metformin (Hu et al., 2016).

Experimental evidence suggests that the JNK/PI3K/Akt signaling pathway is involved in myocardial ischemia/reperfusion injury in diabetic rats. Salvianolic acid A, a water-soluble phenolic acid isolated from the root of Dan Shen, shows an anti-apoptotic effect and improves cardiac function following ischemia/reperfusion injury through the JNK/PI3K/Akt pathway in this model. Pretreatment with SP600125 and salvianolic acid A decreases the p-JNK levels, increases the p-Akt levels, improves cardiac hemodynamics, reduces infarct size and lactate dehydrogenase release, increases sarco/endoplasmic reticulum Ca^2+^ ATPase (SERCA) type 2a activity, decreases Bax and cleaved caspase-3 expression levels, and increases Bcl-2 expression and Bcl-2/Bax ratio in diabetic rats with ischemia/reperfusion injury (Chen Q. et al., 2016). Salvianolic acid A exhibit stronger free radical scavenging capacity and attenuated oxidative stress (Li et al., 2016; Zhu et al., 2017) that could decrease activation of ASK1 and JNK (Figure 2).

### Summary on pharmacological modulation of JNK activity

Promising small molecule agents which exert neuroprotective and cardioprotective effects due to their direct inhibition of JNK activity comprise SP600125, AS601245, IQ-1S, and SR-3306 (Table 1). These compounds control the biological activity of JNK at the level of phosphorylation and dephosphorylation (Carboni et al., 2004, 2005, 2008; Gao et al., 2005; Guan et al., 2005; Murata et al., 2012; Schepetkin et al., 2012; Li et al., 2015; Atochin et al., 2016). Notably, IQ-1S, being a NO-donating oxime and a JNK inhibitor, exerts dual effects both contributing to neuroprotection (Atochin et al., 2016).

The modes of indirect inhibition of JNK activity are quite diverse and precise mechanisms of their protective effects are often insufficiently understood. SU3327 is an inhibitor that attenuates upstream JNK signaling rather than the kinase activity (Jang and Javadov, 2014). Propyl gallate reduces the immunoreactivity of JNK and p38 MAPKs and their phosphorylated forms perhaps due to its antioxidant properties (Wen et al., 2006). D-retro-inverse protein D-JNKI-1 blocks JNK substrate (Borsello et al., 2003; Wen et al., 2006; Wiegler et al., 2008). Penehyclidine hydrochloride downregulates JNK/p38MAPK pathway (Shu et al., 2016) probably due to its cholinergic antagonism. Exendin-4 inhibits phosphorylation of the stress kinases including JNK and is not selective (Kim et al., 2017). Adhesion receptor antagonist, anfibatide, inhibits toll-like receptor 4 (TLR4)/JNK/caspase-3 pathway (Luo et al., 2017). This agent is currently studied in phase II clinical trial for acute coronary syndrome. MAPK phosphatase-1 mediates inactivation of JNK when xanthine oxidase and xanthine dehydrogenase activities are attenuated by the pretreatment with febuxostat (Khan et al., 2017). Febuxostat exerts cardioprotective effects in experimental animals, subjected ischemia/reperfusion, but, according to FDA, this agent shows an increased risk of heart-related death in patients.

Precise molecular mechanisms of modulation of JNK signaling in ischemia/reperfusion remain unclear for many agents showing neuroprotective potential. The list of such agents with poorly understood mechanistic basis includes, but is not limited, by the following: butylphthalide, atorvastatin, CGRP, neuregulin-1β, and a microbial-origin alkaloid K252a.

In summary, researchers have developed and experimentally studied different approaches including administration of small molecules directly blocking JNK activity as well as the proteins and peptides of sophisticated design indirectly interfering with diverse elements of the JNK-associated pathways. To progress from bench to bedside, these approaches and agents require thorough preclinical and, if relevant, clinical investigation to assess their efficacy, safety, and tolerability. Cardioprotective effects of known medications such as anesthetics and NO donors are also sometimes associated with attenuation of JNK activity, which opens the way to create new agents with safe profiles and verified cardioprotective and neuroprotective efficacy based on precursors with known molecular structures. Pharmacological modulation of JNK signaling may be beneficial in reperfusion events common in transplantation, coronary artery disease, cardiac surgery, traumatic injury, and stroke.

## JNKs as potential therapeutic targets

In the past two decades, JNKs have generated interest as potential therapeutic targets for the prevention and treatment of ischemic heart and brain injury (Irving and Bamford, 2002). JNKs are involved in the pathogenesis of stroke, myocardial infarction, diabetes, atherosclerosis, Alzheimer's disease, Parkinson's disease, tumor growth, inflammatory diseases, congestive heart failure and myocardial hypertrophy (Farrokhnia et al., 2005; Waetzig and Herdegen, 2005; Bode and Dong, 2007; Johnson and Nakamura, 2007; Javadov et al., 2014; Ma et al., 2016). Inhibition of JNKs could influence the pathogenesis of these diseases. However, the diversity of physiological properties of JNKs and cross-talk of JNK-dependent pathway with other signaling systems do not allow for systemic use of the non-specific inhibitors affecting the activity of all three isoforms (JNK1, JNK2, and JNK3). Although, several JNK inhibitors with acceptable pharmacokinetics (Gehringer et al., 2015) are currently available, a complete non-specific inhibition of these JNK isoforms would be inappropriate in the treatment of these diseases. However, selective downregulation of individual JNK isoforms (first of all, JNK3 expressed in the brain) or targeting the specific molecular domains of JNK-dependent cascades involved in pathological signal transduction may be promising (Waetzig and Herdegen, 2005; Messoussi et al., 2014). A search for highly selective and nontoxic inhibitors of JNK and JNK pathway continues in order to identify agents with high therapeutic potential. Since JNK3 is expressed in the brain and the heart, the development of selective inhibitors for this isoform may be promising and therapeutic efficacy of new compounds should be studied in appropriate ischemia/reperfusion models. A new inhibitor of JNK IQ-1S (11*H*-indeno[1,2-b]quinoxalin-11-one oxime sodium salt) represents such a high-interest candidate compound. IQ-1S has high affinity to JNK3 and protects the brain after experimental stroke in rats (Atochin et al., 2016). Importantly, pharmacological modulation of JNKs must take into account the presence of comorbidities where JNK could also play an important role. Indeed, diabetic hyperglycemia worsens the ischemic damage to the brain and is associated with JNK phosphorylation in cortical neurons in the experiment (Farrokhnia et al., 2005; Ma et al., 2016). Even in the presence of moderate ischemia, JNK activation is observed on a contralateral side in hyperglycemic rats (Farrokhnia et al., 2005). Cardioprotective and neuroprotective effects of new selective JNK inhibitors, including IQ-1S, should be further explored in the models of ischemia and reperfusion in comorbid animals.

## Author contributions

MS researched literature, wrote the manuscript and contributed to discussion. YA wrote the manuscript, edited the manuscript and contributed to discussion. EA-V reviewed, edited the manuscript and contributed to discussion. IS wrote the manuscript, reviewed, edited the manuscript and contributed to discussion. DA designed, reviewed, edited the manuscript and contributed to discussion.

### Conflict of interest statement

The authors declare that the research was conducted in the absence of any commercial or financial relationships that could be construed as a potential conflict of interest.
